# Generalized DNA Barcode Design Based on Hamming Codes

**DOI:** 10.1371/journal.pone.0036852

**Published:** 2012-05-17

**Authors:** Leonid V. Bystrykh

**Affiliations:** Section Stem Cell Biology, European Research Institute on the Biology of Ageing, University Medical Center Groningen, University of Groningen, Groningen, The Netherlands; University of Otago, New Zealand

## Abstract

The diversity and scope of multiplex parallel sequencing applications is steadily increasing. Critically, multiplex parallel sequencing applications methods rely on the use of barcoded primers for sample identification, and the quality of the barcodes directly impacts the quality of the resulting sequence data. Inspection of the recent publications reveals a surprisingly variable quality of the barcodes employed. Some barcodes are made in a semi empirical fashion, without quantitative consideration of error correction or minimal distance properties. After systematic comparison of published barcode sets, including commercially distributed barcoded primers from Illumina and Epicentre, methods for improved, Hamming code-based sequences are suggested and illustrated. Hamming barcodes can be employed for DNA tag designs in many different ways while preserving minimal distance and error-correcting properties. In addition, Hamming barcodes remain flexible with regard to essential biological parameters such as sequence redundancy and GC content. Wider adoption of improved Hamming barcodes is encouraged in multiplex parallel sequencing applications.

## Introduction

Multiplex deep sequencing is a very powerful approach whenever relatively small DNA fragments should be sequenced within a big number of samples. Instead of analyzing those samples one at a time, DNA samples can be mixed together and sequenced in one run using modern high throughput sequencing machines. This approach requires specific sequence tags that allow finding and identifying the address of any sequence in the mixture and assigning it back to the original sample. Considering that deep sequencing is noisy [1], [2] it imposes several requirements on the tag design. On the one hand those tags should be relatively short to save most of the space to the sample sequencing, on the other hand those tags should be substantially different from one another to prevent cross mutation of sample tags into each other, which correspondingly will cause a cross contamination of the samples. Further, tags might contain some code for fast determination of whether it is authentic or mutated. Finally, tag designs must be flexible to satisfy biological requirements that could be imposed depending on the nuances of the application: such as GC content, sequence redundancy, presence of palindromes etc.

Every tag design relies on the simple combinatorial rule that, with a given number of bases *q* and defined length of the sequence *d,* the total number of combinations is *q^d^*. This predetermines a minimal required length of the tag and generates enough barcoded primers for all samples. The difference in approaches resides in the way of selecting barcodes out of all possible combinations. A literature research revealed a great diversity of selection approaches. The first DNA barcodes were probably designed by random selection, for instance by Bonaldo et al. [3]. Later the problem was dealt with by introducing thorough selection principles out of all possible sequences of a given length [4]–[6]. In some cases, unfortunately, selection principles were not revealed [7]–[9]. Commercially available barcoded primers from Illumina (https://icom.illumina.com/download/summary/ATZRuMiBPkukcRQOJ792Xg) and Epicentre (http://www.epibio.com/pdftechlit/312pl1110.pdf) belong to this group as well. Finally, some proportion of designs (not all of them were aimed at barcodes generation) used not only well defined strategies for the selection of DNA oligonucleotides, but also used elements of coding theory [10]–[15]. All those designs appeared several decades after the pioneering works of Shannon [16], Hamming [17], Reed and Solomon [18] and Levenshtein [19], who established the basics of the coding principles as well as correction of errors in corresponding code words. Considering such a variety of approaches, using coding principles or not, one can argue that since code-containing and code –free barcodes are equally popular, it proves that coding theory is not strictly required for such barcode designs. This is partially true: an invariant property of the DNA barcodes is not a coding principle, but sequence difference between those barcodes. Measures of such differences are known in coding theory as either Hamming or Levenshtein distance, and it is a built-in component of error-correcting codes. In the case of code-free designs, this distance must be achieved using an alignment algorithm and by counting mismatches. Although minimal distance can be achieved by various restrictive algorithms or by simple hand-picking, one is never sure that the best solution has been found. An analysis by alignment is computationally intense, and it often requires custom scripts especially for analysis of short tags. As I will demonstrate here, the benefit of using coding theory is that one can achieve a better result with less computational effort. As a bonus it will retain the benefit of error detection and correction without use of alignment. Considering constraints of size and the subject area, I will focus on linear codes, based mostly on the design of Hamming [17], while not discussing edit-metric codes. This paper aims to provide relatively easy and ready-made examples, to be used by molecular biologists whenever they need to select their own list of tags suited to their application in order to achieve the best possible result.

## Materials and Methods

### Scripts and Analysis

All Hamming codes were made with scripts written in VBA for Excel 2010, some scripts are also duplicated in Python for Gnumeric (http://projects.gnome.org/gnumeric/) spreadsheets. Barcodes generated by others were downloaded when available. All sets of barcodes were re-analyzed in spreadsheet programs mentioned above for minimal distance, GC and sequence redundancy. A decoder script for quaternary codes utilizes Hamming decoder principle (to estimate position of the error), in addition the script contains estimation of the error type and consequently correct code value. This part relies on the modulo operation, which can be of two types: one is “continuous modulo” which counts from negative infinity (in Python and in excel mod function), another one is “zero-flipping modulo”, which takes zero point as the reference (such function is provided in Excel VBA scripts). Some comments on this issue are given by Guido van Rossum (http://python-history.blogspot.com/2010/08/why-pythons-integer-division-floors.html). The decoding algorithm described here relies on a continuous modulo function.

### General Parameters of Codes

Each code contains data bits *d* that in the simplest case represents all possible combinations of given bits, digits or any letters ordered by some principle (alphabetic, low-high etc.); *d* therefore stands for a numeric counter or an index. Note that code-free tags contain only these types of bits (letters, bases). Coded words also contain control or parity bits *p* to detect and possibly correct errors whenever they occur. Therefore for each code word the total length, *n*, will be *n = d+p*. In case of a DNA barcode design it is relevant to define coding redundancy, namely *n/d* (which is the reciprocal of a code rate), this parameter measures the compactness of the design, it is convenient for comparison of different coding strategies. In addition one would be interested in a total possible number of code words, which should be in agreement with experimental design. Depending on the size of the alphabet, *q*, the coding capacity will be *q^d^*, which is 4*^d^* for DNA code, or 2*^d^* for binary code. Parity bits do not extend the number of possible combinations. Importantly, those parity bits add to the differences between codes. To measure those differences the Hamming distance, *d_min_*, can be used. For two code words *c_1_* and *c_2_* of equal length, the number of differing positions is denoted as *d(c_1_,c_2_)*. For instance *d*(“AGC”, “AGT”) = 1*; d*(“ATT”, “AAA”)* = *2*; d*(“TAG”, “GTA”) = 3. Codes with the minimal distance *d_min_ = *2*t+*1 will be able to correct *t* substitution errors [17]. Therefore, in order to correct one or more substitution errors the Hamming distance should be 3 or higher.

Because we deal with DNA, a few more general parameters are essential, namely sequence redundancy, in this paper denoted as *Seq_r_*, it is the size of the longest uninterrupted repetition of the same base in a given tag. For instance *Seq_r_*(“TAAAAC”) = 4. In some cases GC content might be important, especially in microarray applications, because it defines strength of interactions between complementary DNA fragments. Those parameters can also be manipulated during the selection of the coding strategy, otherwise remained uncontrolled. Through the whole manuscript we will aim for a DNA tag length of 6–8 bases as it is most commonly used in the literature.

### General Concept of Hamming Codes

This coding system is unique in its compactness regarding numbers of possible tags generated with a minimal distance of 3 and higher as well as for its algorithm that corrects substitution errors. Briefly, a Hamming code is a binary code constructed from data bits interrupted by parity bits at every 2^n^ position. Parity bits are used for checksum function over different subsets of the data bits, allowing the identification of substitution errors [17]. Hamming used a rather elaborate checksum scheme: the 1^st^ parity bit checks every odd position of the code word starting from the 1^st^ position, the second parity bit checks consecutive pairs of bits starting with the 2^nd^ position and interval of 2 bits, the 3^rd^ bit will check 4 bits in a row starting from position 4 and interval 4, and so on. The whole reason for this system is to have simple error detection algorithm that operates in a binary code only. The classic version of the Hamming code has a length of 7 bits composed of 4 data and 3 parity bits, denoted as Hamming(7,4) code. In a context of DNA coding we might need a binary code with an even number of bits. For this case Hamming suggested adding extra parity position at the end of the code word to check all bits in the word. From this perspective the Hamming(7,4) code can be extended to Hamming(8,4), consequently Hamming(15,11) will be extended to the Hamming(16,11) code. Details of the code design are provided in the Supplementary File S1.

## Results

### Linear Binary-quaternary Code Conversion

Every quaternary symbol (A,C,G,T) can be encoded as a binary word of a length of two bits, for instance using an alphabetical order “A” will be encoded as 00, “C” as 01, “G” as 10, and “T” as 11. With this conversion scheme Hamming binary codes will be translated into a nucleotide sequence by converting every two consecutive bits into the quaternary DNA code. Using the Hamming (16,11) code the 8-base tags can be created as shown in table 1. In binary format such code provides *d_min_* = 4, therefore it should be capable of a single bit error correction and double bit error detection (for details of definitions see Methods). An advantage of this approach is that this encoding –decoding scheme is simple and the error correction algorithm stays intact as created by Hamming. Coding capacity is exceptionally high. However, two aspects of this approach are problematic: since we deal with real DNA, one must consider errors occurring with the DNA sequence, not bits. Each base is 2 binaries, the distance between bases can be either 1 bit (mutation of A to C is 00 to 01, or G to T is 10 to 11) but also two bits, such as A to T conversion is a 01 to 10 mutation. As a result this approach has a serious flaw, namely real minimal distance when converted from binary code into DNA tags is only 2 bases. Hamady et al [14] used such an approach, mistakenly assuming that if Hamming (16,11) code has 5 parity bits it will be sufficient to correct all errors occurring at the DNA level. They further restricted the original Hamming set to those words that generate barcodes with GC content in a range of 40–60% and allowed a sequence redundancy up to 2 bases. Further, Erlich et al [20] restricted this set by selecting every 4^th^ barcode from the Hamady’s list. Potential reduction of the set can increase the minimal distance (http://www.ee.unb.ca/cgi-bin/tervo/hamming.pl). However, direct inspection of selected tags provided by Hamady et al [14] and Erlich et al [20] revealed that this sub-selection still has a *d_min_* = 2 bases. Therefore, despite the authors claim, this set is not error correcting.

**Table 1:** Linear conversion of the Hamming(16,11) code into DNA sequence.

Decimalcounter	Binary data counter	Hamming code	Linear translation into DNA sequence
0	00000000000	0000000000000000	AAAAAAAA (00,00,00,00,00,00,00,00,)
1	00000000001	1101000100000011	TCACAAAT (11,01,00,01,00,00,00,11,)
2	00000000010	0101000100000100	CCACAACA (01,01,00,01,00,00,01,00,)
3	00000000011	1000000000000111	GAAAAACT (10,00,00,00,00,00,01,11,)
4	00000000100	1001000100001000	GCACAAGA (10,01,00,01,00,00,10,00,)
5	00000000101	0100000000001011	CAAAAAGT (01,00,00,00,00,00,10,11,)
6	00000000110	1100000000001100	TAAAAATA (11,00,00,00,00,00,11,00,)
7	00000000111	0001000100001111	ACACAATT (00,01,00,01,00,00,11,11,)
8	00000001000	0001000100010001	ACACACAC (00,01,00,01,00,01,00,01,)
9	00000001001	1100000000010010	TAAAACAG (11,00,00,00,00,01,00,10,)
10	00000001010	0100000000010101	CAAAACCC (01,00,00,00,00,01,01,01,)
11	00000001011	1001000100010110	GCACACCG (10,01,00,01,00,01,01,10,)
12	00000001100	1000000000011001	GAAAACGC (10,00,00,00,00,01,10,01,)
13	00000001101	0101000100011010	CCACACGG (01,01,00,01,00,01,10,10,)
14	00000001110	1101000100011101	TCACACTC (11,01,00,01,00,01,11,01,)
15	00000001111	0000000000011110	AAAAACTG (00,00,00,00,00,01,11,10,)
16	00000010000	1100000100100000	TAACAGAA (11,00,00,01,00,10,00,00,)

Note: Data bits are intercepted by parity bits at every 2^n^ position. Full list of codes are provided in a supplementary File S1.

### Linear Conversion with Overlap

Because linear conversion of the Hamming code shown above is flawed by the nature of the errors, we can check for alternative conversions. The following approach is based on the Hamming(7,4) code which can be used only for small sets of barcodes. The first steps are identical to the linear binary coding, but conversion of binaries to quaternary code occurs by reading two consecutive bits in 1 bit step frame. In this approach, the sequence 001011 will be read as {00, 01, 10, 01, 11}. Thus, a sequence of the length of *n* bits will generate DNA codes of the length *n-1*. Table 2 represents all possible barcodes using Hamming(7,4) code. An advantage of this code is that it acquires extra possibilities of error correction since every binary position is double checked. The *d_min_* = 3 both in a binary and quaternary formats. Hamming(8,4) code made by adding 1 extra parity bit will generate quaternary *d_min_* = 4. Thus, such a coding scheme restores *d_min_* yet shrinks the coding capacity of the set. The sequence redundancy is quite high, which makes such a set unpractical, especially for machines that use pyrosequencing techniques [1]. This parameter can be improved by adding a randomizing function to the sequence, which will for instance invert the bit values every time when two consecutive bits in Hamming code are identical. For conversion into nucleotide sequence we take one bit from the Hamming code sequence and one bit from the randomizer sequence, step size 1 bit. The results are presented at Table 2. The resulting code still holds *d_min_* = 3, *Seq_r_* = 1. However, one can see that the generated sequences are full of simple dimer repeats. Although the idea is useful, all error correcting codes operating in a binary format show quite low coding capacity. One way to improve this is by increasing barcode length to at least *n* = 9. However, a better way is to switch to the quaternary codes.

**Table 2:** Hamming(7,4) codes read with overlap or randomizer.

Decimal counter	Binary data counter	Hamming (7,4)	Conversion	Randomizer	Conversion with randomizer	Hamming (8,4)	Converted
**0**	**0000**	0000000	AAAAAA	0101010	ACACACA	00000000	AAAAAAA
**1**	**0001**	1101001	TGCGAC	0111100	GTCTCAG	11010010	TGCGACG
**2**	**0010**	0101010	CGCGCG	0000000	AGAGAGA	01010101	CGCGCGC
**3**	**0011**	1000011	GAAACT	0010110	GACACTG	10000111	GAAACTT
**4**	**0100**	1001100	GACTGA	0011001	GACTGAC	10011001	GACTGAC
**5**	**0101**	0100101	CGACGC	0001111	AGACTCT	01001011	CGACGCT
**6**	**0110**	1100110	TGACTG	0110011	GTCAGTC	11001100	TGACTGA
**7**	**0111**	0001111	AACTTT	0100101	ACAGTGT	00011110	AACTTTG
**8**	**1000**	1110000	TTGAAA	0100101	GTGACAC	11100001	TTGAAAC
**9**	**1001**	0011001	ACTGAC	0110011	ACTGACT	00110011	ACTGACT
**10**	**1010**	1011010	GCTGCG	0001111	GAGTCTC	10110100	GCTGCGA
**11**	**1011**	0110011	CTGACT	0011001	AGTCAGT	01100110	CTGACTG
**12**	**1100**	0111100	CTTTGA	0010110	AGTGTCA	01111000	CTTTGAA
**13**	**1101**	1010101	GCGCGC	0000000	GAGAGAG	10101010	GCGCGCG
**14**	**1110**	0010110	ACGCTG	0111100	ACTCTGA	00101101	ACGCTGC
**15**	**1111**	1111111	TTTTTT	0101010	GTGTGTG	11111111	TTTTTTT

Each base is read from 2 bit code word, overlap: read 2 consecutive bits from Hamming code with 1 bit step. Randomizer: read 1 bit from Hamming code and 1 bit from randomizer. Both versions have *D_min_* = 3. Hamming(8,4) shows *D_min_* = 4.

Note: randomizer flips bit value in case if 2 consecutive bits in Hamming code are identical.

### Linear Codes in a Quaternary Format

**Figure 1:**
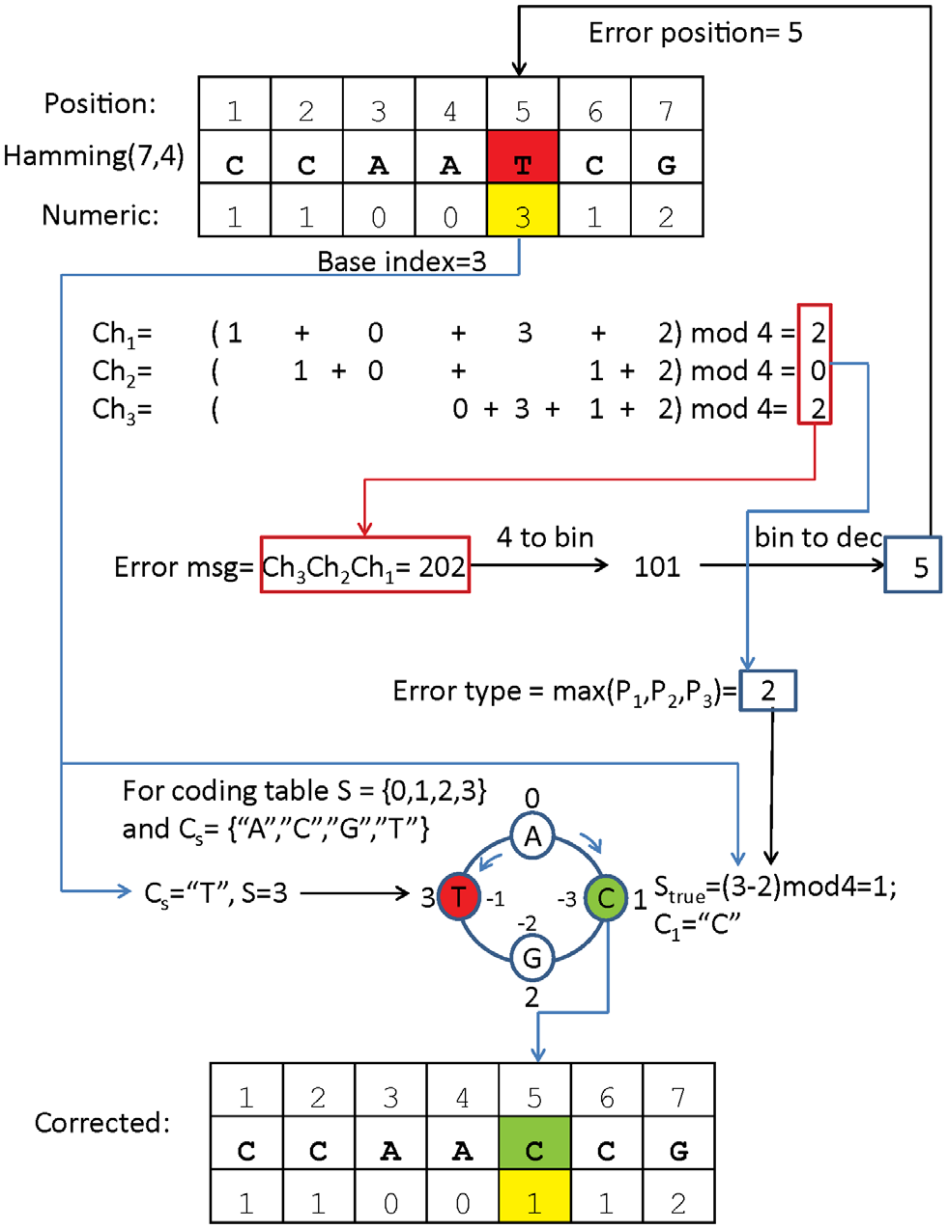
A concept of Hamming error correction in quaternary format. A 7-base sequence is indexed by position and value of each base is provided. With those values checksums are calculated and possible error is detected (in the given example “T” is an error). *Max(Ch_i_)* = 2 gives the type of the error, sequence Ch_3_,Ch_2_,Ch_1_ = 202 is transformed to binary 101 (with the rule: if *Ch_i_>0* then *Ch_i_ = 1*), which is equal to decimal 5. This defines position of the error. Since the value at erroneous position is 3 (for *C_s_* = ”T” *S* = 3), the correct value should be 3−2 = 1. For *S* = 1, *C_s_* = ”C”. Thus, the barcode should be corrected at the position 5, the correct base is “C”. Note when calculating correct base: if *S_true_*<0 then use “the wheel rule” (−3 is 1, −1 is 3, −2 is 2), which can be often (not always!) replaced by *mod 4* operation. In short: *S_true_* =  *(erroneous base value - error type) mod 4*.

The whole elegance of Hamming coding system resides in detecting and correcting errors. The positioning of parity bits is made in such way that when corresponding checksums are put together in one row, they indicate the position of the error in binary format. In principle, Hamming codes can be redefined in a quaternary alphabet thus excluding code conversion. In programming languages, parity check is performed by using modulo function. This function finds the remainder of division of one number by another. For binary code *mod* 2 is used, correspondingly *mod* 4 is used for quaternary code. Bases A,C,G,T will be encoded as 0,1,2,3 correspondingly. The original Hamming error correction principle can be easily adjusted to quaternary code (or any other metrics). The outline is given in Fig. 1. For example, in a Hamming(7,4) code when an error occurs, 1 of the 3 checksums will become non-zero, when put together in a row, Ch3,Ch2,Ch1 will show the position of the error in binary format. For instance binary 010 will report error in the position 2, binary 011 will report error in the position 3 (000 stands for no errors). In binary code the error type is not specified since it is either of two states. In quaternary format when calculating checksum over the bases values, the occurring error will generate checksum values from 1 to 3. This value will be used to identify the correct base relatively to the base produced by error. In addition to this an extra step will be taken, namely converting all non-zero checksums to 1, which will restore the original Hamming error position detection algorithm. An advantage of such an approach is that instead of converting whole codes we only convert the decoding algorithm. Details of such a quaternary Hamming code correction are given in a Supplementary File S2. Note that this decoding algorithm is not limited to the quaternary codes only.

By preserving the Hamming decoding system, quaternary Hamming codes have a much greater coding capacity due to the larger capacity for data counters. The coding capacity of quaternary Hamming(7,4) is 4^4^ = 256 barcodes, which by size would satisfy most of the publications so far. It can be further expanded and reduced according to the requirements of the experiment. Hamming(7,4) code can be reduced to 6-bases code, the coding capacity will be reduced to 64 codes. An example of 6-base long quaternary codes is given in a Table 3. Adding extra parity bases (the same way as in binary code) to Hamming(7,4) will not affect the coding capacity, however it will increase minimal distance from 3 to 4 bases. Full lists of such codes are given in supplementary. The coding efficiency can be further increased by adding more data bases to the code. For instance a Hamming(9,5) code will have the coding capacity 4^5^ = 1024 words. This code is truly error correcting and by size is comparable to the set reported by Hamady et al [14] Hamming(10,6) will give 4^6^ = 4096 words and so on (see also supplementary Files S2 and S3). To my knowledge such version of coding has not been reported in the literature.

**Table 3:** Examples of quaternary Hamming encoded barcode sequences.

Decimal counter	Quaternary data counter	Hamming(6,2)	Conversion	Hamming (6,3)	Conversion
0	000	000000	AAAAAA	000000	AAAAAA
1	001	300311	TAATCC	030301	ATATAC
2	002	200222	GAAGGG	020202	AGAGAG
3	003	100133	CAACTT	010103	ACACAT
4	010	331001	TTCAAC	300310	TAATCA
5	011	231312	GTCTCG	330211	TTAGCC
6	012	131223	CTCGGT	320112	TGACCG
7	013	031130	ATCCTA	310013	TCAACT
8	020	222002	GGGAAG	200220	GAAGGA
9	021	122313	CGGTCT	230121	GTACGC
10	022	022220	AGGGGA	220022	GGAAGG
11	023	322131	TGGCTC	210323	GCATGT
12	030	113003	CCTAAT	100130	CAACTA
13	031	013310	ACTTCA	130031	CTAATC
14	032	313221	TCTGGC	120332	CGATTG
15	033	213132	GCTCTG	110233	CCAGTT

Note: Hamming(6,3) code is incomplete, full set can be found in the supplementary File S2.

### Optimization of Sequence Redundancy

One might notice that all designs mentioned above are not restrictive to the G+C content or sequence redundancy. Table 4 summarizes general properties of some quaternary Hamming codes (as well as other reported tag sets). As one could notice from all previously shown tables, some DNA sequences generated by Hamming codes (e.g. the very first tag) are completely redundant. Such codes should be identified by measuring redundancy and discarded from use in real biological applications. Stringency of filtering depends on the application and sequencing chemistry. Roche 454 pyrosequencing chemistry for instance, imposes serious restriction on this parameter. Solexa platforms and their recent upgrades seem to be very robust in reading homopolymers. In addition, since all high throughput sequencers are tuned up to analyze statistically randomized DNA sequences, it is desirable to have well randomized tags as well. Therefore it is important to identify and eliminate most extreme deviations from random. Although it can be potentially done algorithmically, it is also easy to eliminate it post-algorithmically by simply measuring this parameter and apply a filtering limit. To optimize this parameter Hamady et al [14] used linear Hamming code system followed by removing all “bad” tags by scanning all of them post algorithmically. Meyer et al [4] and Parameswaran et al [5] also used elaborate restrictive approach using code-free barcodes.

**Table 4:** Comparison of commercially available and quaternary Hamming based barcodes.

Barcode set name	Set size	Dmin	GC frequencies									Sequence redundancy							
			0	1	2	3	4	5	6	7	8	1	2	3	4	5	6	7	8
6-mers																			
Epicentre set	12	3–4	0	0	3	8	1	0	0			5	7	0	0	0	0		
H4(6,2)	16	4	1	0	6	0	9	0	0			2	10	2	1	0	1		
H4(6,2) filtered	12	4	0	0	6	0	6	0	0			2	10	0	0	0	0		
H4(6,2) filtered GT transposed	12	4	0	0	0	0	12	0	0			2	10	0	0	0	0		
H4(6,2) filtered AC transposed	12	4	0	0	12	0	0	0	0			2	10	0	0	0	0		
Illumina set	48	2–3	1	1	13	20	8	5	0			17	26	3	2	0	0		
Craig et al., 2008	48	2	4	0	20	16	0	8	0			0	32	13	0	3	0		
H4(6,3)	64	3	1	3	18	26	9	3	4			21	31	8	3	0	1		
H4(6,3) filtered	57	3	0	3	17	26	8	3	0			21	29	7	0	0	0		
7-mers																			
H(7,4)	256	3	2	14	42	70	70	42	14	2		76	60	68	32	12	4	4	
H(7,4) AC transposed	256	3	16	0	0	112	112	0	0	16		76	60	68	32	12	4	4	
H(7,4) AC transposed filtered	224	3	0	0	0	112	112	0	0	0		72	56	64	24	8	0	0	
8-mers																			
H(8,4)	256	4	2	0	56	0	140	0	56	0	2	76	32	88	20	28	8	0	4
H(8,4) AG transposed	256	4	16	0	0	0	224	0	0	0	16	76	32	88	20	28	8	0	4
H(8,4) AG transposed filtered	224	4	0	0	0	0	224	0	0	0	0	72	32	80	16	24	0	0	0
H(16,11)	2048	4	31	0	383	0	1216	0	384	0	32	325	1166	447	73	30	1	1	3
Hamady et al., 2007	1544	2	0	0	0	0	1544	0	0	0	0	536	1008	0	0	0	0	0	0
Erlich et al., 2009	385	2	0	0	0	0	385	0	0	0	0	122	263	0	0	0	0	0	0

Note: H stands for binary Hamming codes, H4 stands for quaternary Hamming codes.

### Optimizing GC Content

For optimization of the GC content previously published strategies can be applied [12], [13]. These schemes however, operate in a binary format, therefore for short sequences coding efficiency is low. In fact, those “bad” sequence redundant tags are also extreme in GC content. Therefore if Seq_r_ -based filtering is applied to those barcodes, they would be eliminated. The rest of the code words show peculiar multimodal distribution of GC frequencies (Table 4). In agreement with the notion of Hamady et al [14] the order of bases coding has an effect on the GC content. When we encode the barcode using alphabetically ordered bases, for S = {0,1,2,3} C_s_ = {A,C,G,T}, a quaternary Hamming(8,4) code will generate 4 sequences having either all or none of G or C bases, further there will be 112 barcodes with either 2 or 6 G+C bases. The remaining 140 barcodes will have exactly half of the bases strong or weak. If we transpose A and G in the original coding table, C_s_ = {G,C,A,T}, then a number of 50% GC containing barcodes will increase from 112 to 224, which is significant improvement in equalizing the GC content across the barcode set. Note that this effect will persist in Hamming codes of every length, yet the distribution of frequencies will vary.

### 6-base Long Codes

Since Illumina and Epicentre commercialized primers containing 6-base barcodes it is worthwhile to have a closer look at their product. Basic properties are already listed in a table 3. As one can see both Illumina and Epicentre seemingly use random selection of barcodes. Those barcodes show a variable minimal distance of 2–3 and 3–4 bases correspondingly. The GC content varies in a range 0–5 for Illumina and 2–4 for Epicentre. Sequence redundancy varies from 2–4 in Illumina tags, and in Epicentre tags it is Seq_r_ = 2 in all tags. Both sets can be successfully challenged by quaternary Hamming(6,3) and Hamming(6,2) codes. Only 2–4 barcodes should be excluded from the code set which exceed the threshold for GC or sequence redundancy. GC content can be made more uniform by using transposed coding table: GT transposition will result in uniform GC content of 4, and AC transposition will yield GC content of 2 uniformly in all barcodes. Other parameters such as coding capacity and minimal distance will be better than in corresponding commercial sets.

At the end, one can see that classical Hamming code adapted to the quaternary coding format is very efficient tool to generate barcodes for multiplex sequencing applications. Only a few tags need to be removed due to sequence redundancy, and GC variations can be optimized by finding the most suitable base –code conversion table. However, every optimization step will come with a cost of reduced coding capacity or increased coding redundancy. Yet it will retain the advantage of the code detection and correction of errors and robust minimal distance.

## Discussion

In biological literature the barcode (a.k.a. bar code or bar-code) stands for a variety of DNA sequences, which can be natural (in case of molecular evolution studies) or artificial (vector libraries, microarrays, multiplexing). By its origin the barcode became public due to the introduction of the Universal Product Code more than 50 years ago. From that time on, barcodes, as familiar to the general consumer society encode commercially available product IDs using visual geometrical symbols. Importantly, this code contains data and parity digits, as well as extra digits for word positioning. When applied to molecular biology, it is appropriate to draw an analogy between the printed stripes and DNA bases. In my opinion, a barcode without a coding part is a sequence tag or DNA oligonucleotide etc. It is ironic to observe a wide range of tags named “barcode”, with substantial variations in their coding qualities or missing the coding component completely. Commercially available barcoded primers from Illumina and Epicentre also contribute to the overall confusion: they are code-free, most likely designed with random generator, and the only rationale behind their design is being different (LB, personal inquiry). A major drawback of random tag design is that none of the essential parameters, *d_min_*, *Seq_r_*, GC%, etc, can be properly controlled. Instead, each of those parameters should be verified in separate protocol to remove failed cases from the list. Note that while *Seq_r_* and GC% are intrinsic tag properties, a *d_min_* parameter belongs to the group of tags. Therefore randomly synthesized tags will have fluctuating differences within the group depending on the length of the tag and number of tags in a group. A process of tag selection to fit to the required minimal distance can be time consuming, since each tag should be cross compared with all other tags in the group. Their designs could be easily replaced by Hamming (6,3) or Hamming (6,2) quaternary codes (Tables 3, 4) providing more robust minimal distance, minimize sequence redundancy and achieve more tags than presented both by Epicentre and Illumina together. Early designs of barcodes used for DNA microarray provide interesting algorithms with sufficient minimal distance and error correcting capacity [11], [13], yet they were made for longer, microarray type of oligonucleotides and were left unused in recent publications. Currently, multiplex parallel sequencing is steadily growing in its diversity and extent of application. Recent applications demand the concurrent sequencing of several thousand samples in parallel. [14], [20]. Under these circumstances, the highest quality barcodes are critical to the success of the analysis. Flawed barcode design can have dire consequences such as erroneously assigned sequences and cross contamination of data sets. Two recent publications, although used a coding concept [14], [15], both provided barcode sets with a *d_min_* = 2, which is not enough to correct single substitution errors. Minimal distance is important not only to correct errors, but also to protect samples against cross contamination and keep the noise in the sequencing data at a minimum. It is concerning that those flawed designs remained undetected while used by others. Here I show that possible applications of the Hamming codes in DNA tagging are not fully explored. With a few examples in this paper one can see that by employing different coding systems we can obtain sets of primer tags of different sizes, length and error correcting capacity. Whereas binary Hamming code based tags proposed by Hamady et al., [14] will fail with 1/3 of all base substitution errors, quaternary Hamming code based tags will correct all of them.

It is fair to say, that Hamming codes are sensitive to insertion/deletion (indel) mutations which cannot be corrected with the existing algorithm. The major source of errors in Solexa type machines are substitutions [2]. Therefore linear codes are quite suitable for this application. Pyrosequencing based instruments, like 454 GS-FLX by Roche, make indel type of errors when reading homopolymer sequences. This, however, can be circumvented by controlling sequence redundancy as it was demonstrated here. This will eventually improve the performance of linear codes again. Although true indel errors cannot be detected or corrected by linear codes, their contribution to the sequencing noise is at least 10 times lower compared to substitutions [2]. An advantage of using linear codes is in its simplicity as well as coding capacity, which is much better than edit metric codes (codes capable of correcting single indels). There is always a tradeoff between size of a tag set, the tag’s length and minimal distance. It is up to the researcher to decide first how large a multiplexing experiment would be designed, and then choose a proper coding scheme. Although it is not always important to correct for errors, a fast identification of true non-mutated barcodes will be provided by coding/decoding algorithms. A wider range of options described here should further stimulate specialized barcode designs, improve the quality of the data and suit better to the requirements of the real biological experiment. The serious obstacle in such interdisciplinary field is proper translation of coding theory into biological application, without this many potentially progressive theories will be left unused.

When this manuscript was in a process of reviewing I was suggested to respond on the recent publication by Krishnan et al., [21]. Authors used binary BCH codes, which upon linear binary-quaternary conversion yielded DNA barcodes 8 or 16 bases long with *d_min_* 4 or 7 bases respectively. These are robust designs that surely contribute to the field of synthetic barcodes design (e.g. see comparison of errors recovery in supplementary File S4). Importantly for this paper, the authors make two remarkable statements. First, authors referred to the design by Hamady et al. [14] and stated, “though Hamming codes possess low decoding complexity, they have a minimum distance of 3, albeit very low. Indeed, it can be shown that these barcodes cannot guarantee the recovery from even one sequencing error”. It is true for Hamady’s design and not true for Hamming codes in general. As it was shown here Hamming codes can be implemented in different ways, therefore it depends on the conversion scheme and accompanying factors. Secondly, the authors also stated “no efficient and systematic decoding algorithms exist for decoding quaternary codes that are of interest in DNA barcoding”. If it is true, this is the first paper where a decoding algorithm for the quaternary code is provided. Besides, it works with code of any alphabet size.

## Supporting Information

File S1 Hamming binary codes.
(XLS)

File S2 Hamming quaternary codes. This file contains scripts for barcodes analysis: minimal distance can be measured using script “align”. Properties of barcode series can be re-analyzed using script “properties”. Frequencies of the properties are summarized with script “counts”.
(XLS)

File S3 Gnumeric Python files zipped. It contains H4_74.gnumeric file with quaternary Hamming (7,4) codes generated with H4code74_GNU.py script, decoder.gnumeric file contains fragment of H4(7,4) code for generation and correction of errors, using random_GNU.py and decoder_GNU.py scripts correspondingly. H4code74.r and H4code74_decoder.r are two R-scripts written by Erik Zwart.
(DOCX)

File S4 Poisson calculator. It illustrates performance of different linear codes.
(XLS)
