# Supplementary material for: Generalized DNA Barcode Design Based on Hamming Codes
Source: PLoS One. 2012 May 17;7(5):e36852. doi: 10.1371/journal.pone.0036852 (PMC3355179; doi:10.1371/journal.pone.0036852)
Supplement: File S3 — Gnumeric Python files zipped. It contains H4_74.gnumeric file with quaternary Hamming (7,4) codes generated with H4code74_GNU.py script, decoder.gnumeric file contains fragment of H4(7,4) code for generation and correction of errors, using random_GNU.py and decoder_GNU.py scripts correspondingly. H4code74.r and H4code74_decoder.r are two R-scripts written by Erik Zwart. (DOCX) [file pone.0036852.s003.docx]

Supplementary file S3 contains H4code74_GNU.py script to generate H4(7,4) code, random_GNU.py generates random errors in the H4(7,4) code and and decoder_GNU.py scripts corrects those errors correspondingly. Note that all those codes operate within Gnumeric spreadsheet. Gnumeric files are available on request. H4code74.r and H4code74_decoder.r are two R-scripts written by Erik Zwart.

#script H4code74_GNU.py

# this script generates quaternary Hamming codes

#It requires Gnumeric spreadsheet

import Gnumeric, os

wb=Gnumeric.workbooks()[0] #output in workbook sheet1

s=wb.sheets()[1]

c=s[0,1]

x=1

total=0

p=[0,0,0,0,0,0,0,0]

d=[0,0,0,0,0,0,0,0]

base=['A','C','G','T']

s[0,0].set_text('Index')

s[1,0].set_text('Data')

s[2,0].set_text('HQ code')

s[3,0].set_text('Barcode')

while x<257:

H74=[]

s[0,x].set_text(str(x-1)) #decimal counter#

data=d[1],d[2],d[3],d[4]

s[1,x].set_text(str(data)) #set data values

#count parity bits.

p[1]=(4-(d[1]+d[2]+d[4])%4)%4

p[2]=(4-(d[1]+d[3]+d[4])%4)%4

p[3]=(4-(d[2]+d[3]+d[4])%4)%4

#generates binary Hamming sequence

H74.extend([p[1],p[2],d[1],p[3],d[2],d[3],d[4]])

s[2,x].set_text(str(H74))

#generates sequence out of H74 code and base coding table

seq=''

mylen=len(H74)

w=0

while w<mylen:

seq=seq+base[H74[w]]

w+=1

# print seq

s[3,x].set_text(str(seq)) #writes the barcode

d[4]+=1 # counter for the next HQ code

if d[4]==4:

d[4]=0

d[3]+=1

if d[3]==4:

d[3]=0

d[2]+=1

if d[2]==4:

d[2]=0

d[1]+=1

x+=1

#execfile('/home/leonid/Desktop/Pytrhon/Hamming/H4code74_GNU.py')

#end of script---------------------------------------------------------------------------------------------

#Script random_GNU.py

#This script is designed for gnumeric file

#decoder.gnumeric

#it will read H4 codes in the column 2

#(note row and column count begins with 0)

#and create mutated barcodes in the column 3

import Gnumeric, os

wb=Gnumeric.workbooks()[0]

s=wb.sheets()[0] #output in workbook sheet1

#c=s[0,1]

# this is my random numbers generator

#based on the random bytes generator

si= os.urandom(100)

num = int(si.encode('hex'), 16)

L=list(str(num))

base=list('ACGT')

for i in range(1,30):

#read barcodes in the 2nd column

tag=s.cell_fetch(1,i).get_value()

if tag==None:

break

b=list(tag)

#generate random base at random position

myvalue=(int(L[i])*10+int(L[i+1]))%7

mybase=(int(L[i])*10+int(L[i+1]))%4

#replace by random base

b[myvalue]=base[mybase]

#generate mutated barcode

s[2,i].set_text(b[0]+b[1]+b[2]+b[3]+b[4]+b[5]+b[6])

# type there the line below to execute (uncomment first)

#execfile('/home/leonid/Desktop/Python/Hamming/random_GNU.py')

#end of script---------------------------------------------------------------------------------------------

#Script decoder_GNU.py

#this script is written for the gnumeric file decoder.gnumeric

#it reads mutated barcodes from column 3 and further

# corrects quaternary Hamming codes

import Gnumeric, os

wb=Gnumeric.workbooks()[0] #output in workbook sheet2

s=wb.sheets()[0]

c=s[0,1]

x=1

total=0

p=[0,0,0,0,0,0,0,0]

d=[0,0,0,0,0,0,0,0]

base=['A','C','G','T']

#check range of barcodes

for x in range (1,30):

mystring=s.cell_fetch(2,x).get_value()

if mystring==None:

break

#clear previous data

for y in range (1,7):

s[2+y,x].set_text('')

H74=list(mystring)

HQ74=[]

#check each position in the barcode

for n in H74:

#convert bases to values

for i in range(0,4):

if n==base[i]:

HQ74.append(i)

#show values

s[3,x].set_text(str(HQ74))

#checksums

p[1]=(HQ74[0]+HQ74[2]+HQ74[4]+HQ74[6])%4

p[2]=(HQ74[1]+HQ74[2]+HQ74[5]+HQ74[6])%4

p[3]=(HQ74[3]+HQ74[4]+HQ74[5]+HQ74[6])%4

#find the maximum

p_max=max(p[1],p[2],p[3])

#convert checksums in binaries

for i in range (0,4):

if p[i]>0:

p[i]=1

s[4,x].set_text(str(p_max))

if p_max>0:

#concatenate in reverse

# bin_pos=bin(p[3]+p[2]+p[1])

bin_pos=str(p[3])+str(p[2])+str(p[1])

print bin_pos

s[5,x].set_text(bin_pos)

err_pos=int(bin_pos,2)

s[6,x].set_text(str(err_pos))

#wrong value min error type %4 should give the right answer

corr_value=(HQ74[err_pos-1]-p_max)%4

s[7,x].set_text(str(corr_value))

s[8,x].set_text(H74[err_pos-1] +' to '+ base[corr_value] + ' at pos '+ str(err_pos))

H74[err_pos-1]=base[corr_value]

s[9,x].set_text(str(H74[0]+H74[1]+H74[2]+H74[3]+H74[4]+H74[5]+H74[6]))

#use python console inside gnumeric to execute this script

#execfile('/home/leonid/Desktop/Python/Hamming/decoder_GNU.py')

#end of script---------------------------------------------------------------------------------------------

#Script H4code74.r

# modification of H4code74_GNU.py script

# Generate quaternary Hamming codes

# number of possible barcodes 4^4=256

# set data matrix

# p1 p2 d1 p3 d2 d3 d4 (d=data, p=parity)

data = matrix(nrow=(4^4),ncol=7)

colnames(data) = c("p1","p2","d1","p3","d2","d3","d4")

# set dummy counter

c = 1;

# begin creating barcodes

for (d1 in 0:3) {

for (d2 in 0:3) {

for (d3 in 0:3) {

for (d4 in 0:3) {

# set the parity bits

p1 = (4-((d1+d2+d4)%%4))%%4

p2 = (4-((d1+d3+d4)%%4))%%4

p3 = (4-((d2+d3+d4)%%4))%%4

# put data sequence into data matrix

data[c,c(1:7)] = c(p1,p2,d1,p3,d2,d3,d4)

c = c+1

}

}

}

}

# create the actual barcodes out of the H74 coding table (data)

barcodes = data

barcodes[barcodes == 0] <- "A"

barcodes[barcodes == 1] <- "C"

barcodes[barcodes == 2] <- "G"

barcodes[barcodes == 3] <- "T"

# print numeric encoded barcodes

data

# print DNA barcodes

Barcodes

#end of script---------------------------------------------------------------------------------------------

#Script H4code74_decoder.r

# modification of decoder_GNU.py script

# Corrects quaternary Hamming codes

# set of valid hamming codes

valid_codes = c("GGCTAAC","TTCAAAA","GAACTCT","CCAGTTA","GCCGACC","GCAGTAT","CGATTGA","AGATTCC")

# set of mutated hamming codes (correspond with the above valid codes) error at position 1,2,3,4,5,6,7 and none

error_codes = c("TGCTAAC","TACAAAA","GAGCTCT","CCATTTA","GCCGCCC","GCAGTGT","CGATTGG","AGATTCC")

# convert to numeric

# in example below the first code is called from error_codes "TGCTAA" (error_codes[1])

x = as.matrix(rbind(unlist(strsplit(error_codes[1],""))))

# convert characters to numeric

x[x == "A"] <- 0

x[x == "C"] <- 1

x[x == "G"] <- 2

x[x == "T"] <- 3

# calculate the checksums

p1 = (as.numeric(x[1])+as.numeric(x[3])+as.numeric(x[5])+as.numeric(x[7]))%%4

p2 = (as.numeric(x[2])+as.numeric(x[3])+as.numeric(x[6])+as.numeric(x[7]))%%4

p3 = (as.numeric(x[4])+as.numeric(x[5])+as.numeric(x[6])+as.numeric(x[7]))%%4

# find the maximum

p_max = max(p1,p2,p3)

# 'convert' checksums in binary

if (p1>0) {p1=1}

if (p2>0) {p2=1}

if (p3>0) {p3=1}

# concatenate in reverse

bin_pos = paste(p3,p2,p1, sep="")

# the error position

err_pos = bin2dec(bin_pos)

# calculate the correct value of the error

# wrong value min error type %%4 should give the right answer

corr_value = (as.numeric(x[err_pos])-p_max)%%4

# remember to load this function before calling it.

bin2dec <- function(x) {

x <- as.character(as.numeric(x))

b <- as.numeric(unlist(strsplit(x, "")))

pow <- 2 ^ ((length(b) - 1):0)

sum(pow[b == 1])

}

# corr_value = correcting value

# 0,1,2,3 == A,C,G,T

corr_value

# err_pos = error position

err_pos
